# Preoperative subcategorization based on magnetic resonance imaging in intrahepatic cholangiocarcinoma

**DOI:** 10.1186/s40644-023-00533-2

**Published:** 2023-02-13

**Authors:** Yuyao Xiao, Changwu Zhou, Xiaoyan Ni, Peng Huang, Fei Wu, Chun Yang, Mengsu Zeng

**Affiliations:** 1 Department of Radiology, Zhongshan Hospital, Fudan University, No. 180 Fenglin Road, Xuhui District, Shanghai, 200032 China; 2 Shanghai Institute of Medical Imaging, No. 180 Fenglin Road, Xuhui District, Shanghai, 200032 China; 3 Department of Cancer Center, Zhongshan Hospital, Fudan University, Shanghai, China

**Keywords:** Cholangiocarcinoma, Liver neoplasms, Magnetic resonance imaging, Diagnosis criteria

## Abstract

**Background:**

Appropriate preoperative identification of iCCA subtype is essential for personalized management, so the aim of this study is to investigate the role of MR imaging features in preoperatively differentiating the iCCA subtype.

**Methods:**

Ninety-three patients with mass-forming intrahepatic cholangiocarcinoma (iCCA, 63 small duct type and 30 large duct type) were retrospectively enrolled according to the latest 5th WHO classification (mean age, males vs. females: 60.66 ± 10.53 vs. 61.88 ± 12.82, 50 men). Significant imaging features for differentiating large duct iCCA and small duct iCCA were identified using univariate and multivariate logistic regression analyses, and a regression-based predictive model was then generated. Furthermore, diagnostic performance parameters of single significant imaging features and the predictive model were obtained, and corresponding receiver operating characteristic (ROC) curves were subsequently presented.

**Results:**

The univariate analysis showed that tumor in vein, arterial phase hypoenhancement, intrahepatic duct dilatation, lack of targetoid restriction and lack of targetoid appearance in T2 were predictors of large duct type iCCA. Arterial phase hypoenhancement, intrahepatic duct dilatation and lack of targetoid restriction were independent predictors for large duct type iCCA in multivariate analysis. The regression-based predictive model has achieved the best preoperative prediction performance in iCCA subcategorization so far. The area under the ROC curve of the regression-based predictive model was up to 0.91 (95% CI: 0.85, 0.98), and it was significantly higher than every single significant imaging feature.

**Conclusions:**

Arterial phase hypoenhancement, intrahepatic duct dilatation and lack of targetoid restriction could be considered reliable MR imaging indicators of large duct type iCCA. MR imaging features can facilitate noninvasive prediction of iCCA subtype with satisfactory predictive performance.

**Supplementary Information:**

The online version contains supplementary material available at 10.1186/s40644-023-00533-2.

## Introduction

Intrahepatic cholangiocarcinoma (iCCA) is the second most common primary liver cancer, which is exhibiting increasing mortality [1, 2], and it can be further subcategorized into the large duct type and the small duct type based on the origin of the cells (intrahepatic large bile ducts and peribiliary glands for the large duct type, and small bile ducts and bile ductules/hepatic progenitor cells for the small duct type) in the latest 5th WHO classification. In addition, there are quite different clinicopathological and molecular features and prognosis between the two subtypes [3–9].

To date, it has been verified that patients with small duct iCCA tend to exhibit better survival outcome and less recurrence than those with large duct iCCA [3, 7–9]. Moreover, isocitrate dehydrogenase (IDH)-1 and − 2 mutations and fibroblast growth factor receptor (FGFR) fusion, which are restricted to the small duct type [3], can be targeted by currently available drugs [10], thus providing new and promising therapies for terminal inoperable cases. Therefore, appropriate preoperative identification of iCCA subtype is essential for personalized management, but this identification now primarily relies on the pathological findings.

Magnetic resonance imaging (MRI) may have a role in subcategorizing iCCA subtype preoperatively and noninvasively, as small duct iCCA mainly appears as an anatomical location of the peripheral liver and a growth pattern of mass-forming (MF) type [3–5]. However, previous studies have shown that approximately 30% of small duct type iCCA occurred in the perihilar area, and that both iCCA subtypes could manifest as the MF type on imaging presentations [11, 12]. There have also been some studies that have attempted to identify differences between the two subtypes in terms of enhancement patterns, biliary abnormalities and CT imaging features [11, 13–15]. Recently, Park et al. [16] has evaluated the role of gadoxetic acid-enhanced MRI in subtype classification of iCCA; although its capability of liver function assessment, it risks misdescribing enhancement pattern, especially in arterial phase. To our knowledge, few studies have comprehensively investigated which extracellular contrast agent-based MR features can potentially differentiate iCCA subtype preoperatively.

Thus, we aimed to investigate the role of Gadobutrol-based MR imaging features in preoperatively differentiating the iCCA subtype.

## Materials and methods

This study was the Institutional Review Board approved, and the requirement for informed consent was waived for a retrospective design.

### Patient selection

From December 2019 to December 2021, 171 patients who were pathologically diagnosed with MF iCCA were consecutively identified according to the 5th WHO classification. A total of 162 patients who met the following criteria were included in the analysis: (1) number of iCCA lesions less than 5; and (2) contrast enhancement MR imaging being performed within 2 weeks before surgery. Among the 162 patients who met the inclusion criteria, 69 patients were excluded because of (1) insufficient quality for MR images (*n* = 2); (2) preoperative treatments before MR imaging (*n* = 13); and (3) incomplete pathological data (*n* = 54). Finally, 93 patients with 101 iCCA lesions (3 patients with 2 lesions, 1 patient with 3 lesions and 1 patient with 4 lesions) were enrolled in this study, with an average age of 61.3 ± 11.6 years old, and 50 (52.6%) patients were male. Among the patients with more than one iCCA lesion, the lesions with the largest diameter were assessed.

### Clinicopathological data evaluation

Relevant clinical information of iCCA patients was retrospectively collected from the medical records, including (a) demographic data (age, sex); (b) hepatitis B virus (HBV) infection status; and (c) serum tumor biomarkers (levels of serum AFP and serum CA 19-9).

According to pathologic reports, 93 patients were categorized into two groups by iCCA subtype (the large duct type and the small duct type) according to the latest 5th WHO classification definition [3]. Large duct iCCA originates in intrahepatic segmental branches of the biliary tree, and shows a ductal or tubular pattern comprised of mucin-positive tall columnar cholangiocytes. Small duct iCCA originates in the bile duct smaller than segmental branches, and shows a tubular pattern comprised of mucin-negative cuboidal cholangiocytes. In addition, the pathological findings of the lesions, such as Ki-67 index, microvascular invasion (MVI, presence or absence), and tumor size (≤ 2 cm, 2-5 cm and ≥ 5 cm) were also evaluated.

### MR examinations

MR images were acquired via a 1.5 T MR scanner (uMR 560, United Imaging Healthcare) with a 24-channel coil. Routine plain-scan MR imaging included T1-weighted in-phase and out-of-phase sequences, transverse T2-weighted fast spin-echo sequence and diffusion-weighted imaging (DWI) with b values of 0, 50, and 500 s/mm2. Dynamic imaging was performed with a T1-weighted fat-suppressed sequence. Gadobutrol (Gadavist; Bayer HealthCare) was intravenously administered at a rate of 2 ml/s for a dose of 0.1 mmol/kg. When the contrast agent reached the ascending aorta, arterial phase acquisition was automatically triggered, and the portal venous phase (70-90 s) and delayed phase (160-180 s) were acquired subsequently. All of the detailed parameters of each sequence have been previously reported [17].

### Image analysis

All MR images were independently viewed by two experienced radiologists in abdominal imaging analysis (C.Y. and C.W.Z., with 14 and 12 years of experience respectively). Both radiologists were blinded to the clinicopathological data and laboratory tests, but they knew that the patients were diagnosed with MF iCCA. When there was any inconsistency between the two observers, they negotiated until a consensus was reached.

The following imaging features of iCCA were investigated on plain-scan MR images: (a) restricted diffusion (presence or absence; targetoid or not), (b) intratumoral hemorrhage, (c) intrahepatic duct dilatation, (d) hepatic capsule retraction and (e) T2-weighted signal intensity (signal intensity compared with spleen; targetoid or not). In addition, the presences or absences of the following features were evaluated on dynamic enhancement images: (A) arterial phase: (a) enhancement patterns (hypoenhancement, non-rim arterial phase hyperenhancement and rim arterial phase hyperenhancement), and (b) corona enhancement; (B) portal venous phase: (c) washout patterns (non-peripheral washout, peripheral washout), (d) enhancing capsule, and (e) tumor in vein; (C) delayed phase: (f) delayed central enhancement. The definition of the MR imaging features mentioned above were detailed in supplementary material.

### Statistical analysis

Statistical analyses were performed by using SPSS 25.0 (IBM) and R software (version 4.1.2). Continuous variables were presented as the mean ± standard deviation (for normal distribution) or median (interquartile range) (for skewed distribution), and were compared by independent-sample t test or Mann-Whitney U test, respectively. Chi-square test or Fisher’s exact test were used to detect differences of categorial variables between the large duct type group and the small duct type group. Following univariable logistic regression analysis, imaging features exhibiting *p* values < 0.05 in univariate logistic analysis were then incorporated into multivariate analysis to identify significant independent predictors for iCCA subtype. Kappa statistics were used to evaluate the interreader agreement in interpretation of images, and were defined as follows: ≤0.20, poor; 0.21–0.40, fair; 0.41–0.60, moderate; 0.61–0.80, substantial; and 0.81–1.00, almost perfect.

The sensitivity, specificity, accuracy, positive likelihood ratio and negative likelihood ratio of the significant imaging findings and regression-based predictive model were calculated, and were then compared by McNemar test. Receiver operating characteristic curves of the single significant imaging findings and predictive model derived from logistic regression analysis for predicting iCCA subtype were constructed, and the corresponding area under receiver operating characteristic (AUC) curves were then obtained with 95% CIs and compared by using delong test. The significance level was considered to be a *p* value less than 0.05.

## Results

### Clinicopathological features

**Fig. 1:**
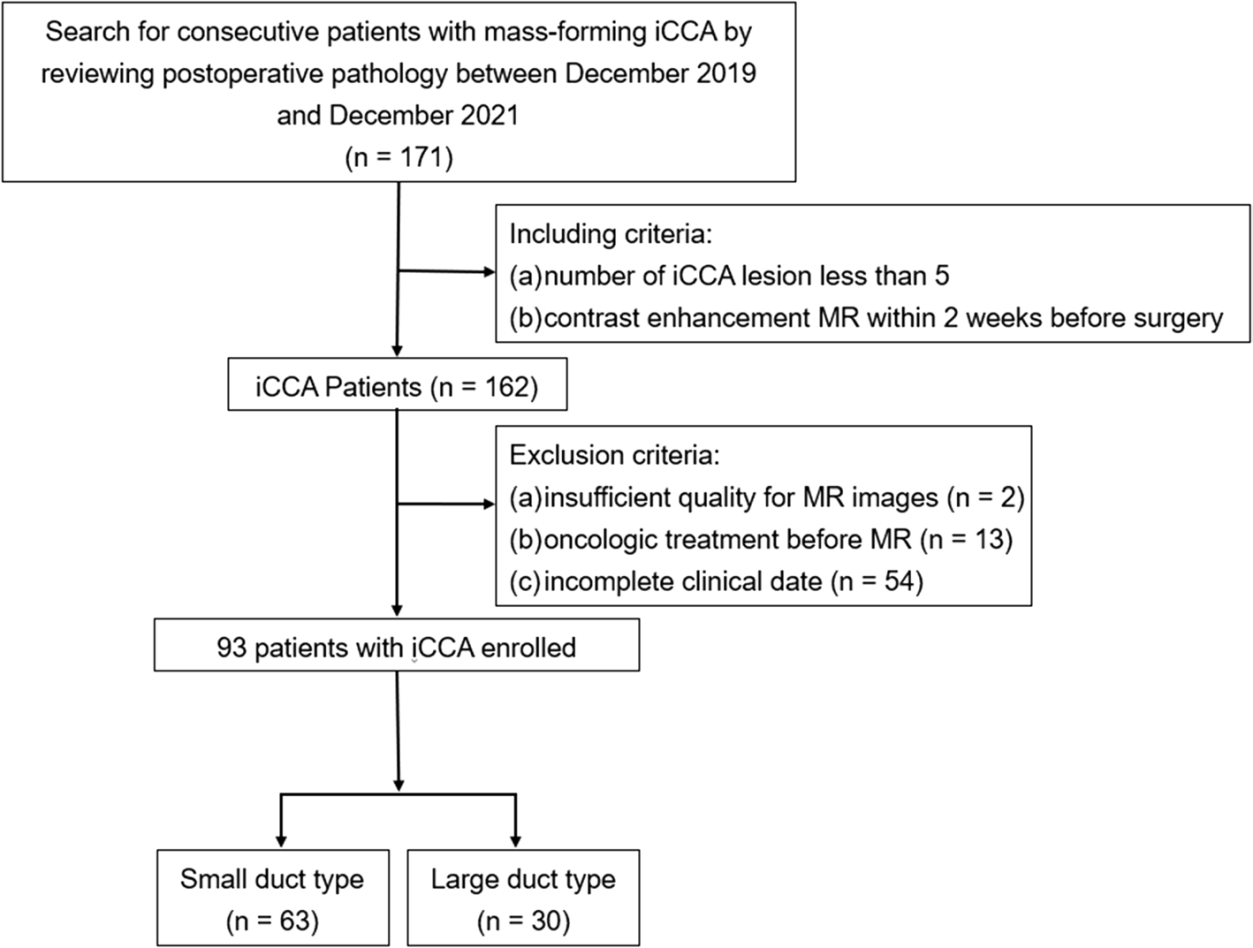
Flowchart of this study cohort. iCCA = intrahepatic cholangiocarcinoma

**Fig. 2:**
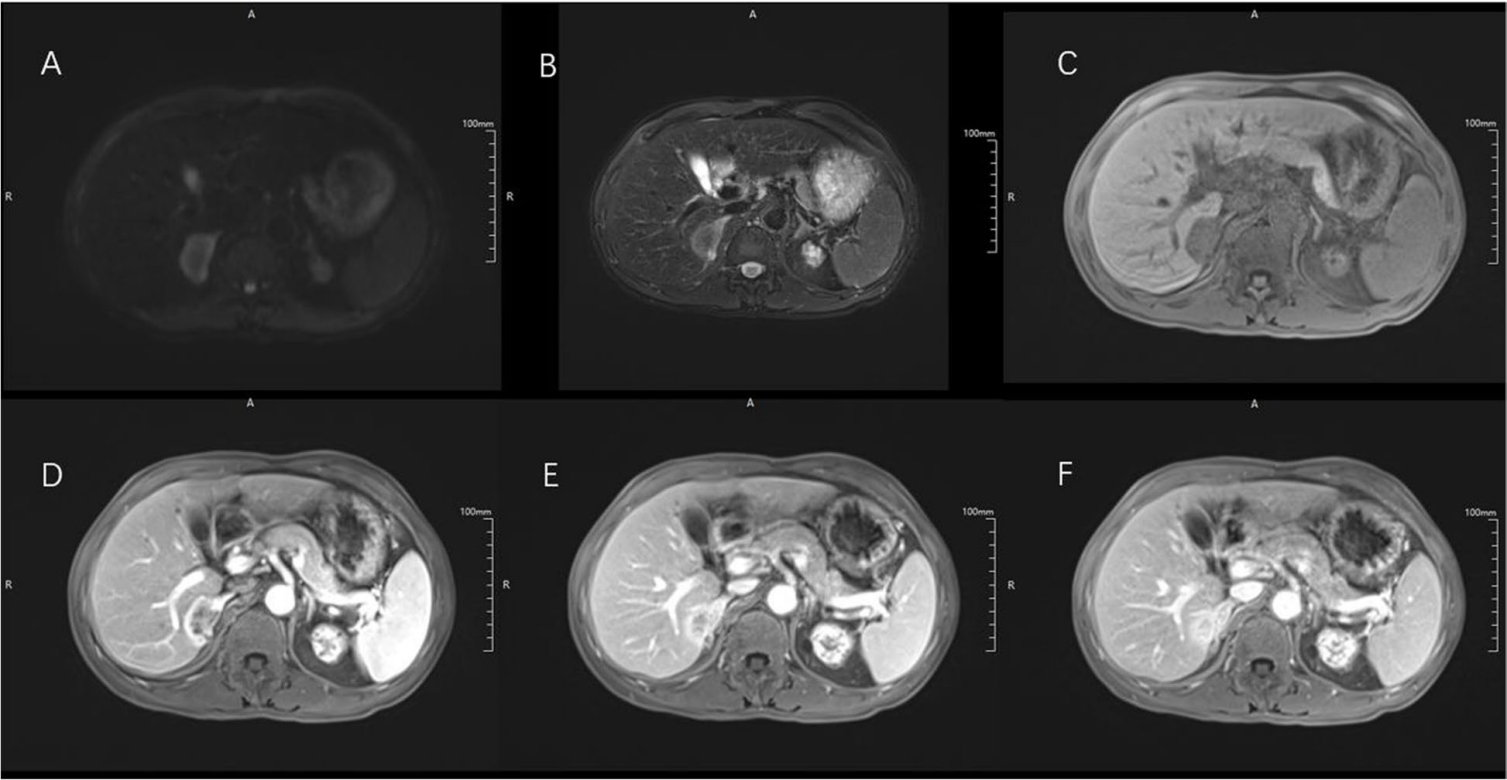
Images in a 48-year-old man with surgically-confirmed small duct type intrahepatic cholangiocarcinoma in the right lobe of the liver. **A** On diffusion-weighted image with b value of 500 s/mm2, the mass shows the targetoid restriction. The mass demonstrated mild-moderate hyperintensity on T2WI (**B**) and hypointensity on T1WI (**C**). On arterial phase (**D**), it showed rim arterial phase enhancement and then showed gradual central delayed enhancement on portal venous phase and delayed phase (**E**, **F**). Also, no appearent intrahepatic duct dilatation was found in this patient

**Fig. 3:**
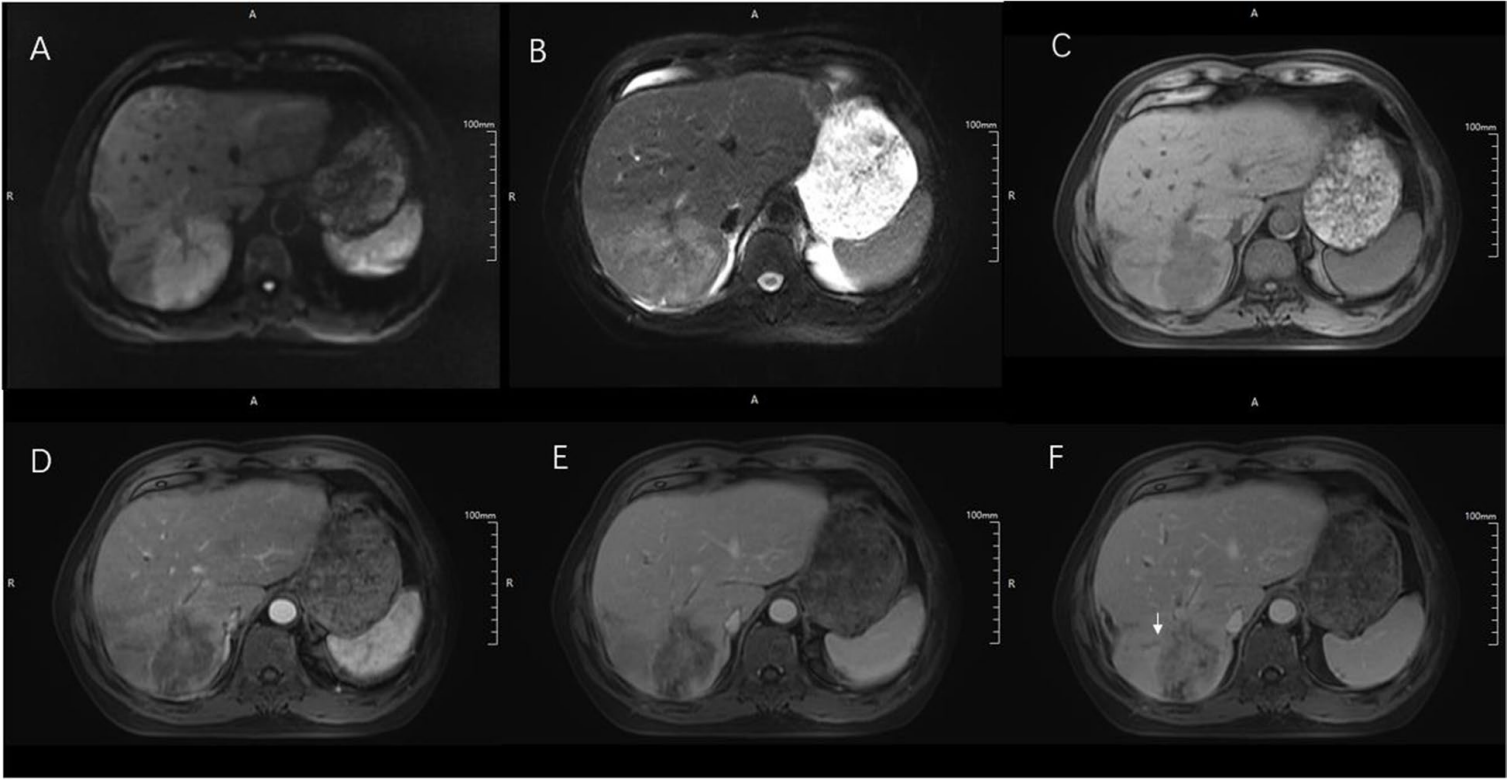
Images in a 69-year-old man with surgically-confirmed large duct type intrahepatic cholangiocarcinoma in the right lobe of the liver. **A** On diffusion-weighted image with b value of 500 s/mm2, the mass shows the homogeneous restriction. The mass demonstrated mild-moderate hyperintensity on T2WI (**B**) and hypointensity on T1WI (**C**). On arterial phase (**D**), it showed no enhancement and then showed gradual central delayed enhancement on portal venous phase and delayed phase (**E**, **F**). Also, intrahepatic duct dilatation was found in this patient around the mass (white arrow)

Among the Ninety-three patients, 63 patients were assigned into the small duct type group while 30 into the large duct type group according to pathologic analyses. Table 1 provides a detailed comparison of the clinicopathological features of the iCCA patients. Patients with large duct type iCCA presented a significantly higher level of CA 19-9 (median, 19.9 vs. 94.5, *p* = 0.001) and Ki-67 index (median, 40 vs. 60, *p* = 0.024). In addition, patients subcategorized into large duct type group were marginally older than those in small duct type group (mean, 59.6 ± 11.9 vs. 64.6 ± 10.8, *p* = 0.053) and exhibited a slightly higher frequency of MVI (13.3% vs. 30.8%, *p* = 0.056). Instead, HBV infection was more common in patients with small duct iCCA (42.9% vs. 20.0%, *p* = 0.031). There were no significant differences in sex, AFP level or tumor size between small duct type and large duct type groups (all *p* > 0.05) (Figs. 1, 2 and 3).

**Table 1:** Clinical and pathological characteristics of patients with iCCA

Characteristics	All (*n* = 93)	Subtype		
		Small duct type (*n* = 63)	Large duct type (*n* = 30)	*p* value
Age (years) ^a^	61.2 ± 11.7	59.6 ± 11.9	64.6 ± 10.8	0.053
Sex (male)	50 (53.8%)	36 (57.1%)	14 (46.7%)	0.344
HBV infection	33 (35.5%)	27 (42.9%)	6 (20.0%)	0.031*
AFP ^b^	2.5 (1.8,3.5)	2.3 (1.8,3.4)	2.8 (1.6,4.1)	0.348
CA19-9 ^b^	26.8 (10.8115.5)	19.9 (9.3,50.3)	94.5 (26.31997.5)	0.001*
Ki-67 index ^b^	40 (30,70)	40 (30,60)	60 (30,80)	0.024*
MVI ^c^	16 (18.6%)	8 (13.3%)	8 (30.8%)	0.056
Tumor size (cm)				0.886
≤ 2	5 (5.4%)	3 (4.8%)	2 (6.7%)	
2-5	43 (46.2%)	30 (47.6%)	13 (43.3%)	
≥ 5	45 (48.4%)	30 (47.6%)	15 (50.0%)	

*iCCA* Intrahepatic cholangiocarcinoma, *HBV* Hepatitis B virus, *AFP* Alpha fetoprotein, *CA 19-9* Carbohydrate antigen 19-9, *MVI* Microvascular invasion

^a^ Data are mean ± standard deviation; ^b^ data are median (interquartile range). Except where labeled, data are numbers of patients, with percentages in parentheses

^c^ data was available in 86 patients

* *p* <  0.05

### MRI characteristics

Interreader agreement for in interpretation of MR features is detailed in Table 2. The presence of targetoid restriction (49.2% vs. 13.3%, *p* = 0.001) and targetoid appearance in T2 (38.1% vs. 6.7%, *p* = 0.002) was significantly higher in the small duct type group than in the large duct type group, whereas arterial phase hypoenhancement (4.8% vs. 30.0%, *p* = 0.001), tumor in vein (6.3% vs. 23.3%, *p* = 0.018) and intrahepatic duct dilatation (17.5% vs. 76.7%, *p* <  0.001) were more common in patients with large duct type iCCA than in those with small duct type iCCA. In addition, rim APHE was slightly more common in small duct iCCA (76.2% vs. 56.7%, *p* = 0.055). No significant difference was found regarding intratumoral hemorrhage, restricted diffusion, non-rim arterial phase peritumoral enhancement (APHE), non-peripheral washout, enhancing capsule, peripheral washout, delayed enhancement or hepatic capsule retraction (all *p* > 0.05) between the small duct type and large duct type groups. The comparisons of MRI characteristics between small duct iCCA and large duct iCCA patients are described in Table 3.

**Table 2:** The inter-reader agreements of imaging features

Imaging Features	κ value	95%CI	*p* value
Intratumoral hemorrhage	0.789	0.506-1.000	< 0.001
Restricted diffusion	NA	NA	NA
Arterial phase hypoenhancement	0.732	0.511-0.953	< 0.001
Non-rim APHE	0.568	0.350-0.786	< 0.001
Non-peripheral washout	0.783	0.548-1.000	< 0.001
Targetoid appearance in T2	0.871	0.761-0.981	< 0.001
Corona enhancement	0.574	0.405-0.742	< 0.001
Enhancing capsule	0.809	0.629-0.989	< 0.001
Tumor in vein	0.755	0.528-0.982	< 0.001
Rim APHE	0.570	0.388-0.752	< 0.001
Peripheral washout	0.647	0.482-0.812	< 0.001
Delayed enhancement	0.625	0.415-0.835	< 0.001
Targetoid restriction	0.638	0.475-0.800	< 0.001
Intrahepatic duct dilatation	0.802	0.682-0.922	< 0.001
Hepatic capsule retraction	0.605	0.442-0.768	< 0.001

*APHE* Arterial phase hyperenhancement

**Table 3:** MR Imaging features of iCCA

Imaging features	Subtype		
	Small duct type (*n* = 63)	Large duct type (*n* = 30)	*p* value
Intratumoral hemorrhage ^a^	2 (3.2%)	2 (6.7%)	0.438
Targetoid restriction	31 (49.2%)	4 (13.3%)	0.001*
Restricted diffusion	63 (100%)	32 (100%)	> 0.999
Arterial enhancement pattern			
Arterial phase hypoenhancement	3 (4.8%)	9 (30%)	0.001
Rim APHE	48 (76.2%)	17 (56.7%)	0.055
Non-rim APHE	12 (19.0%)	4 (13.3%)	0.495
T2-weighted signal intensity			
Diffused iso- / mild-hyperintense	20 (31.7%)	15 (50.0%)	0.089
Diffused marked hyperintense	19 (30.2%)	13 (43.3%)	0.211
Targetoid appearance in T2	24 (38.1%)	2 (6.7%)	0.002*
Peripheral washout	19 (30.2%)	4 (13.3%)	0.079
Non-peripheral washout ^a^	5 (7.9%)	1 (3.3%)	0.372
Delayed enhancement	54 (85.7%)	26 (86.7%)	0.901
Corona enhancement	25 (39.7%)	8 (26.7%)	0.220
Enhancing capsule ^a^	8 (12.7%)	3 (10.0%)	0.706
Tumor in vein ^a^	4 (6.3%)	7 (23.3%)	0.018*
Intrahepatic duct dilatation	11 (17.5%)	23 (76.7%)	< 0.001*
Hepatic capsule retraction	29 (46.0%)	11 (36.7%)	0.394

*iCCA* Intrahepatic cholangiocarcinoma, *APHE* Arterial phase hyperenhancement, *LR* LI-RADS

^a^ data were compared by Fisher’s exact test. *p <  0.05

### Uni−/multivariate analyses for predictive factors

Table 4 summarizes the results of univariate and multivariate analysis for imaging features associated with the iCCA subtype. The univariate analysis showed that tumor in vein, arterial phase hypoenhancement, intrahepatic duct dilatation, lack of targetoid restriction and lack of targetoid appearance in T2 were predictors of large duct type iCCA. Arterial phase hypoenhancement (OR = 8.186, *p* = 0.028), intrahepatic duct dilatation (OR = 37.500, *p* <  0.001) and lack of targetoid restriction (OR = 0.079, *p* = 0.008) were independent predictors for large duct type iCCA in multivariate analysis.

**Table 4:** Uni/Multivariate analysis of predictors for iCCA subpe

Imaging features	Univariate analysis for subtype			Multivariate analysis for subtype		
	*p* value	OR	95% CI	*p* value	OR	95%CI
Intratumoral hemorrhage	0.448	2.179	0.292-16.266			
Restricted diffusion	NA	NA	NA			
Targetoid restriction	0.002*	0.159	0.050-0.508	0.008*	0.079	0.012-0.520
Arterial phase hypoenhancement	0.003*	8.571	2.118-34.688	0.028*	8.186	1.257-53.287
Rim APHE	0.058	0.813	0.162-1.032			
Non-rim APHE	0.497	0.654	0.192-2.228			
Targetoid appearance in T2	0.006*	0.116	0.025-0.532	0.083	0.141	0.015-1.294
Peripheral washout	0.087	0.356	0.109-1.162			
Non-peripheral washout	0.413	0.400	0.045-3.584			
Delayed enhancement	0.901	1.083	0.305-3.847			
Corona enhancement	0.223	0.553	0.197-1.434			
Enhancing capsule	0.707	0.764	0.188-3.112			
Tumor in vein	0.026*	4.489	1.200-16.798	0.606	0.582	0.074-4.567
Intrahepatic duct dilatation	< 0.001*	15.532	5.8343-45.156	< 0.001*	37.500	6.985-201.329
Hepatic capsule retraction	0.395	0.679	0.278-1.657			

*p < 0.05. *iCCA* Intrahepatic cholangiocarcinoma, *OR* Odds Ratio, *95% CI* 95% Confidence interval, *APHE* Arterial phase hyperenhancement, *LR* LI-RADS

### Diagnostic performance

The sensitivity, specificity, accuracy, positive likelihood ratio, negative likelihood ratio and area under receiver operating characteristic curve (AUC) values of significant imaging features and predictive model are presented in Table 5. The specificity of arterial phase hypoenhancement for differentiating iCCA subtype was up to 95.2% whereas the sensitivity of this factor was the lowest (30.0%). In contrast, targetoid restriction achieved the highest sensitivity of 86.7%, as well as the lowest specificity of 57.0%. Among the three imaging features, intrahepatic duct dilatation achieved the highest accuracy (83.9%), with sensitivity and specificity of 76.7 and 82.5%, respectively. The regression-based predictive model achieved satisfactory diagnostic performance with the sensitivity and specificity comparable with targetoid restriction (80.0% vs. 86.7%, *p* = 0.500) and arterial phase hypoenhancement (93.7% vs. 95.2%, *p* > 0.999), respectively. When regarding receiver operating characteristic curves based on the single imaging feature and predictive model for predicting iCCA subtype, the AUC of the predictive model was up to 0.91 (95% CI: 0.85, 0.98), and it was significantly higher than every single imaging feature (all *p* <  0.001) (Table 5).

**Table 5:** Diagnostic performance of the significant imaging features and predictive model

Imaging features	Sensitivity	Specificity	Accuracy	Positive LR	Negative LR	AUC	*P* value ^a^	*P* value ^b^	*P* value ^c^
AP hypoenhancement	30.0% (9/30)	95.2% (60/63)	64.5% (69/93)	6.25	0.74	0.63 [0.50,0.76]	–	< 0.001/< 0.001/0.429	< 0.001/0.008/0.011
Targetoid restriction	86.7% (26/30)	49.2% (31/63)	57.0% (57/93)	1.71	0.27	0.68 [0.57,0.79]	< 0.001/< 0.001/0.429	–	0.250/< 0.001/0.099
Intrahepatic duct dilatation	76.7% (23/30)	82.5% (52/63)	83.9% (78/93)	4.38	0.28	0.80 [0.69,0.90]	< 0.001/0.008/0.011	0.250/< 0.001/0.099	–
Predictive model	80.0% (24/30)	93.7% (59/63)	89.2% (83/93)	12.70	0.210	0.91 [0.85,0.98]	< 0.001/> 0.999/< 0.001	0.500/< 0.001/< 0.001	> 0.999/0.016/< 0.001

*AP* Arterial phase, *LR* Likely ratio, *AUC* Area under curve

^a^ *P* values were obtained by comparing sensitivity/specificity/AUC between AP hypoenhancement and other characteristics. ^b^ *P* values were obtained by comparing sensitivity/specificity/AUC between targetoid restriction and other characteristics. ^c^ *P* values were obtained by comparing sensitivity/specificity/AUC between intrahepatic duct dilatation and other characteristics

## Discussion

The typical imaging feature of MF iCCA is hypoenhancement/rim enhancement in arterial phase followed by gradual delayed enhancement [18–22], and it was able to correctly diagnose over 80% of iCCA cases in the current study. However, in the context of further preoperatively subcategorizing iCCA based on MRI, few imaging features have been extracted to date. Our results demonstrated that arterial phase hypoenhancement, intrahepatic duct dilatation and lack of targetoid restriction could be considered reliable MR imaging indicators of large duct type iCCA. By utilizing these imaging features, we proposed a regression-based predictive model for the iCCA subtype achieving an area under the ROC curve up to 0.91 (95% CI: 0.85, 0.98), with sensitivity of 80.0% and specificity of 93.7%.

It has been demonstrated that small duct iCCA is more frequently associated with the same risk factors as hepatocellular carcinoma (HCC), including viral hepatitis and non-biliary cirrhosis, whereas large duct iCCA shares risk factors with perihilar and extrahepatic cholangiocarcinoma, such as primary sclerosing cholangitis and liver fluke infection [3]; thus, differences in etiology-related carcinogenetic backgrounds may indirectly explain why patients with small duct iCCA tend to exhibit clinical features more like HCC (relatively younger age of morbidity, lower serum CA 19-9 level and higher frequency of HBV infection) in our study. These results were consistent with those of previous studies [3, 9, 23]. Moreover, microvascular invasion (MVI), higher Ki-67 index and tumor in vein (also termed as macrovascular invasion) were considered important predictors of prognosis in various tumors [24–27], so their high prevalence in large duct iCCA conformed to the fact that large duct iCCA exhibits more aggressive pathological features and worse prognosis, as described in the 5th WHO classification [3–8]. Several studies have obtained results similar to our findings [7, 9, 28].

Interestingly, targetoid restriction (49.2% vs. 13.3%, *p* = 0.001), targetoid appearance in T2 (38.1% vs. 6.7%, *p* = 0.002) and rim APHE (76.2% vs. 56.7%, *p* = 0.055) were, significantly or marginally, more prevalent in small duct iCCA in our study. Considering the correlation between imaging and histology, we thus suspected that these results may be attributed to the discrepancy in the arrangement of fibrous stroma. According to previous studies [19, 29, 30], small duct iCCA usually exhibits a kind of arrangement of peripheral tumor cells and central fibrous stroma, whereas diffuse distribution of fibrous stroma at different degrees has mainly been observed in large duct iCCA. For the same reason, it’s explainable that arterial phase hypoenhancement was more common in large duct iCCA, and this result was very consistent with previous studies [12, 13], which claimed that large duct iCCA tends to be of hypovascular, whereas small duct iCCA was often assigned to rim-enhancing group or hypervascular group.

Among the significant variables, intrahepatic duct dilatation was significantly more frequent in the large duct group than in the small duct group, with the highest odds ratio (OR = 33.167, *p* <  0.001) at multivariate logistic analysis. As has been previously mentioned [3], the growth pattern of large duct iCCA predominately comprises a periductal infiltrating pattern or periductal infiltrating with mass forming pattern, which may make bile duct wall thickening and bile duct narrowing more prevalent at tumor sites in large duct iCCA, thus leading to more frequent upper intrahepatic bile duct dilatation in the large duct type.

Arterial phase hypoenhancement was one of the most frequently mentioned imaging features in previous studies [12, 13] and can reliably identify large duct iCCA. In the present study, arterial phase hypoenhancement achieved a high specificity, however, with an unsatisfactory sensitivity. Our results were inconsistent with Fujita’s [12] in diagnostic performance of AP hypoenhancement but similar with Park’s [16], and the relatively small sample size may be the underlying factor explaining this discrepancy. What’s more, the regression-based predictive model derived in our study also showed excellent specificity to subcategorize iCCA subtype preoperatively, but maintained satisfactory sensitivity, which has achieved the best preoperative prediction performance in iCCA subcategorization so far. Therefore, as small duct iCCA tend to exhibit better outcome and targetable gene mutations, the predictive model we proposed may be of paramount significance for both prognostic and therapeutic purposes.

This study has limitations. First, selection bias seemed to be inevitable because of the single-center and retrospective design of the study. Second, our study enrolled patients who were diagnosed with iCCA in the last 2 years, so we were regretfully incapable of following up long enough to evaluate prognostic differences. Third, iCCA patients usually present with advanced disease, but inoperable patients were not included in our study, which may introduce biases. Finally, the sample size, especially the number of large duct type iCCA, was relatively small in our study. Therefore, our findings need to be validated through larger multi-center prospective studies.

## Conclusions

In conclusion, arterial phase hypoenhancement, intrahepatic duct dilatation and lack of targetoid restriction could be considered reliable MR imaging indicators of large duct type iCCA. MR imaging features can facilitate noninvasive prediction of iCCA subtype with satisfactory predictive performance.

## Supplementary Information

**Additional file 1.**

## Data Availability

The datasets used and/or analysed during the current study are available from the corresponding author on reasonable request.

## Abbreviations

iCCA: Intrahepatic cholangiocarcinoma
ROC: Receiver operating characteristic
AUC: Area under curve
APHE: Arterial phase hyperenhancement
