# Supplementary material for: Preoperative subcategorization based on magnetic resonance imaging in intrahepatic cholangiocarcinoma
Source: Cancer Imaging. 2023 Feb 13;23:15. doi: 10.1186/s40644-023-00533-2 (PMC9926776; doi:10.1186/s40644-023-00533-2)
Supplement: Supplementary file 1 — Additional file 1. [file 40644_2023_533_MOESM1_ESM.docx]

| **Imaging features** | **definition** |
| --- | --- |
| intratumoral hemorrhage | a hyperintense area on T1-weighted images, with variable signal intensity on T2-weighted images |
| intrahepatic duct dilatation | intrahepatic duct dilatation within or outside of the lesion |
| hepatic capsule retraction | retraction of hepatic capsular adjacent to the lesion |
| AP hypoenhancement | The observation that demonstrates iso- to hypointesion without a hyperattenuation area in arterial phase |
| nonrim arterial phase hyperenhancement | arterial phase hyperenhancement is not most pronounced in periphery of observation |
| rim arterial phase hyperenhancement | arterial phase hyperenhancement is most pronounced in periphery of observation |
| T2 diffused iso-/mild-hyperintense | T2-weighted signal intensity is equal to or higher than liver parenchyma, but not higher than spleen |
| T2 diffused marked hyperintense | T2-weighted signal intensity is higher than both liver parenchyma and spleen |
| targetoid appearance in T2 | a central area of less intensity, compared to a more hyperintense peripheral area on T2-W sequences. |
| corona enhancement | peri-observational enhancement in late arterial phase or early PVP attributable to venous drainage from tumor |
| nonperipheral washout | visually assessed temporal reduction in enhancement of an observation relative to composite liver tissue from an earlier to a later phase resulting in nonperipheral hypoenhancement on the portal venous or delayed phases |
| peripheral washout | visually assessed temporal reduction in enhancement of an observation relative to composite liver tissue from an earlier to a later phase resulting in peripheral hypoenhancement on the portal venous or delayed phases |
| enhancing capsule | enhancing rim in portal venous phase or delayed phase |
| tumor in vein | Unequivocal presence of enhancing soft tissue in vein |
| delayed central enhancement | central area of progressive postarterial phase enhancement. |
